# Transcriptomic Analysis Identifies Candidate Genes and Gene Sets Controlling the Response of Porcine Peripheral Blood Mononuclear Cells to Poly I:C Stimulation

**DOI:** 10.1534/g3.116.028290

**Published:** 2016-02-29

**Authors:** Jiying Wang, Yanping Wang, Huaizhong Wang, Haifei Wang, Jian-Feng Liu, Ying Wu, Jianfeng Guo

**Affiliations:** * Shandong Provincial Key Laboratory of Animal Disease Control and Breeding, Institute of Animal Science and Veterinary Medicine, Shandong Academy of Agricultural Sciences, Jinan 250100, China; † Key Laboratory of Animal Genetics, Breeding and Reproduction, Ministry of Agriculture, College of Animal Science and Technology, China Agricultural University, Beijing 100193, China

**Keywords:** polyinosinic-polycytidylic acid (poly I:C), peripheral blood mononuclear cells (PBMC), RNA-seq, differentially expressed (DE) genes, pigs

## Abstract

Polyinosinic-polycytidylic acid (poly I:C), a synthetic dsRNA analog, has been demonstrated to have stimulatory effects similar to viral dsRNA. To gain deep knowledge of the host transcriptional response of pigs to poly I:C stimulation, in the present study, we cultured and stimulated peripheral blood mononuclear cells (PBMC) of piglets of one Chinese indigenous breed (Dapulian) and one modern commercial breed (Landrace) with poly I:C, and compared their transcriptional profiling using RNA-sequencing (RNA-seq). Our results indicated that poly I:C stimulation can elicit significantly differentially expressed (DE) genes in Dapulian (g = 290) as well as Landrace (g = 85). We also performed gene set analysis using the Gene Set Enrichment Analysis (GSEA) package, and identified some significantly enriched gene sets in Dapulian (g = 18) and Landrace (g = 21). Most of the shared DE genes and gene sets were immune-related, and may play crucial rules in the immune response of poly I:C stimulation. In addition, we detected large sets of significantly DE genes and enriched gene sets when comparing the gene expression profile between the two breeds, including control and poly I:C stimulation groups. Besides immune-related functions, some of the DE genes and gene sets between the two breeds were involved in development and growth of various tissues, which may be correlated with the different characteristics of the two breeds. The DE genes and gene sets detected herein provide crucial information towards understanding the immune regulation of antiviral responses, and the molecular mechanisms of different genetic resistance to viral infection, in modern and indigenous pigs.

During the past decades, production levels of livestock and poultry have increased considerably in both reproduction and growth performance. However, highly productive animals have difficulty in coping with environmental challenges, and exhibit immunological problems (Prunier *et al.* 2010). In the animal industry, various infectious diseases caused by viral or bacterial pathogens severely limit production efficiency and cause huge economic losses. Consequently, selection of livestock and poultry for increased disease resistance is of major economic importance. The resistance of an individual is largely dependent on its genetic potential of immunocompetence. Previous reports have indicated that considerable variation in resistance to infectious disease exists among individual animals within or between breeds (Reiner *et al.* 2002; Flori *et al.* 2011a). Genetic improvement of the immune capacity of pigs by breeding for general immune capacity could increase the productivity and welfare of pigs (Stear *et al.* 2001; Flori *et al.* 2011b). Thus, a deep understanding of the networks of genes that control porcine immune responses, and that contribute to disease-resistance phenotypes, has become a central research subject in farm husbandry.

In the immune system, whole blood cells, especially peripheral blood mononuclear cells (PBMC), consisting of many types of immune cells that circulate in the whole body, such as lymphocytes, granulocytes, and monocytes/macrophages, constitute one of the first lines of defense of the immune system. Transcriptome profiling of PBMCs throughout immune responses is widely used to dissect pathogenesis and the genetics behind infection or stimulation (Gao *et al.* 2010; Uddin *et al.* 2012; Adler *et al.* 2013), and such studies have demonstrated that measuring the PBMC transcriptome is useful in identifying genes controlling variability of disease resistance in the pig. Thus, a transcriptomic investigation of the molecular portraits underlying individual differences in immune capacity in pigs could represent an important approach to unveiling the genetic basis of disease resistance.

Double-stranded RNA (dsRNA) is not a major constituent of mammalian cells, but many viruses produce it during their replication cycle, as either an essential intermediate for RNA synthesis or a byproduct of viral RNA/DNA synthesis (Jacobs and Langland 1996). Polyinosinic:polycytidylic acid (poly I:C), a synthetic dsRNA analog, can mimic viral infection, and has been demonstrated to have stimulatory effects similar to those of viral dsRNA (Alexopoulou *et al.* 2001; Caskey *et al.* 2011; Cao *et al.* 2013). Poly I:C can serve as a pathogen-associated molecular pattern (PAMP), and induce the activation of the NF-κβ and type I interferon signaling cascades through two major dsRNA sensors, toll-like receptor 3 (TLR3), and melanoma differentiation-associated protein-5 (MDA5) (Alexopoulou *et al.* 2001; Kato *et al.* 2006; McCartney *et al.* 2009).

The domestic pig (*Sus scrofa*) is not only one of the most important meat-producing livestock species worldwide, but is an excellent animal model with which to study various microbial infectious diseases due to its similarity to humans in terms of anatomy, genetics, and physiology (Meurens *et al.* 2012). Evidence indicates that responses to virus infection are different among pig breeds (Vincent *et al.* 2006; Meurens *et al.* 2012), and indigenous pigs are generally more resistant than modern commercial pigs (Halbur *et al.* 1998; Jiang *et al.* 2013). However, the genetic basis and immune mechanism of such differences are still not well understood. In humans, genome-wide expression analyses have been performed on GRE cells (Geiss *et al.* 2001), and PBMCs (Huang *et al.* 2006) to gain a global perspective of *ex vivo* viral infection models. In pigs, using Affymetrix expression microarray, Liu *et al.* (2014) recently conducted transcriptome analysis of whole blood coming from piglets that were chosen based on serum IFN-a levels at 4 hr post poly I:C treatment. Detailed knowledge of the gene expression profiles generated in porcine PBMC with poly I:C stimulation *in vitro* is currently lacking.

The development of high-throughput functional genomics technologies, especially RNA-sequencing (RNA-seq), has enabled detailed analysis of the host transcriptome response to pathogen infection or immunostimulants. To gain a deeper knowledge of the host transcriptional response to poly I:C stimulation, in the present study, using piglets of one modern commercial breed (Landrace) and one Chinese indigenous breed (Dapulian), we cultured PBMC of these piglets, stimulated these cells with poly I:C, and compared their transcriptional profiling using RNA-seq. Additionally, we also compared the gene expression profiles of PBMC to identify gene expression differences between the two breeds. This study describes, for the first time, the PBMC transcriptomic response to poly I:C stimulation in pigs, and provides crucial regulatory information to help understand the immune regulation of antiviral responses, and the molecular mechanisms of different genetic resistance to viral infection in modern and indigenous pigs.

## Materials and Methods

### PBMC isolation and poly I:C stimulation

Six 5-wk-old piglets, selected from one modern commercial breed (Landrace), and one Chinese indigenous breed (Dapulian), were used in the study. All the piglets were raised in the same facility, and had not received any vaccinations except one for classical swine fever (CSF) 21 d after birth. The whole study protocols for collection of blood from the experimental pigs were reviewed and approved by the Institutional Animal Care and Use Committee (IACUC) of Shandong Academy of Agricultural Sciences.

For each piglet, 20 ml blood was collected via venipuncture into a vacutainer tube containing anticoagulant (EDTAK_2_). PBMC were isolated using Ficoll-Hypaque PLUS (GE Healthcare), following the manufacturer’s instructions. In brief, the whole blood was first diluted by an equal volume of phosphate buffer solution (PBS). Then, 20 ml of diluted blood was carefully added on top of 10 ml of Ficoll-Hypaque solution in a 50 ml conical tube and centrifuged at 460 × *g* for 20 min at room temperature. After centrifugation, the middle whitish interface containing mononuclear cells was transferred to a new tube, and washed by PBS followed by centrifugation at 1000 rpm for 10 min twice. Cells were counted using a hemocytometer, and their viability was confirmed by exclusion of the vital dye trypan blue.

PBMC, isolated from every piglet, were diluted into 70 ml RPMI-1640 medium (Hyclone), supplemented with 10% fetal calf serum, 100 IU/ml of penicillin, and 100 μg/ml of streptomycin, to a final cell concentration of ∼2 × 10^6^/ml. The cell suspension was divided into two parts; to one part poly I:C (Sigma-Aldrich) was added to a final concentration of 20 μg/ml (stimulated group), with medium added to the other past as an unstimulated control (control group). Both stimulated and unstimulated PBMC were cultured for 24 hr at 37° with 5% CO_2_. A preliminary study (Wang *et al.* 2015) determined that concentration of 20 μg/ml and a 24-hr time point gave the largest immune response among the three concentrations (10 μg/ml, 20 μg/ml, and 40 μg/ml) and four time points (4 hr, 8 hr, 12 hr, and 24 hr) tested. Finally, we obtained 12 samples, which were divided into four groups: DT (three samples of Dapulian stimulated by poly I:C), DC (three samples of Dapulian cultured 24 hr), LT (three samples of Landrace stimulated by poly I:C), and LC (three samples of Landrace cultured for 24 hr).

### mRNA library construction and sequencing

Total RNA was extracted from each sample using RNAiso Plus (TaKaRa) following the manufacturer’s procedure. The integrity and yield of RNA were measured using a NanoDrop 2000 spectrophotometer (Thermo Scientific), and an Agilent 2100 Bioanalyzer (Agilent Technologies, Santa Clara, CA). RNA of all samples had an RNA integrity number score of ≥8, and rRNA 28S/18S ≥1.6.

For each sample, approximately 5 μg of total RNA was used to isolate poly(A) mRNA using poly-T oligo attached to magnetic beads (Invitrogen). Following purification, mRNA was fragmented into small pieces using divalent cations under elevated temperature. Then, the cleaved RNA fragments were reverse-transcribed to create the final cDNA library in accordance with the protocol from the mRNA-seq sample preparation kit (Illumina, San Diego, CA). The average insert size for the paired-end libraries was 300 bp (±50 bp). Then, for each of the 12 samples, we performed paired-end sequencing with a read length of 100 bp on an Illumina HiSequation 2000.

### Sequencing data processing

Raw RNA-seq data were filtered according to the following criteria: 1) reads containing sequencing adaptors were removed; 2) nucleotides with a quality score lower than 20 were trimmed from the end of the sequence; and 3) reads shorter than 50 were discarded. After the filtering pipeline, high quality clean data were aligned pig reference genome (Sscrofa10.2) downloaded from Ensembl (ftp://ftp.ensembl.org/pub/release-75/fasta/susscrofa/dna/). Alignment was performed by using an improved version of TopHat 2 (Kim *et al.* 2013) with default parameters.

The aligned read files were processed by Cufflinks, which uses the normalized RNA-seq fragment counts to measure the relative abundance of the transcripts. The unit of measurement is fragment per kilobase of exon per million fragments mapped (FPKM), *i.e.*, the number of fragments was normalized by the transcript’s length, and the total yield of the fragments, to ensure accurate quantification of the gene’s expression (Trapnell *et al.* 2010). First, Cufflinks was used to assemble the transcriptome *de novo*, and then Cuffmerge was used to merge all transcripts of samples to generate unique transcripts. The downloaded Ensembl GTF file was passed to Cuffdiff along with the original alignment (SAM) files produced by TopHat. Cuffdiff reestimated the abundance of the transcripts listed in the GTF file, using alignments from the SAM file, and tested concurrently for different expression. Only comparisons with “q value” (FDR adjusted p-value) less than 0.05, log2(fold-change) > 1 or < −1, and status marked as “OK” in the Cuffdiff output, were regarded as showing differential expression.

### Gene functional analysis of differentially expressed genes

Gene ontology (GO) enrichment analysis was performed using the topGO package (version 3.1) (Alexa and Rahnenfuhrer 2010). The classic Fisher method was chosen to obtain the p-values of every term, and false discovery rate (FDR) was used to adjust multiple testing.

A gene set analysis using the Gene Set Enrichment Analysis (GSEA) package was also used to analyze the pattern of differential gene expression between the two groups. We used canonical pathways (CP) of curated gene sets c2 (c2_CP), which contain gene sets collected from the pathway databases (BioCarta, KEGG and Reactome) in the Molecular Signature Database version 4.05 (MSigDB; http://www.broad.mit.edu/gsea/msigdb/msigdb_index.html). During the analysis, the gene set permutation option (1000 times permutation) was conducted to calculate significance and FDR values.

### Validation of mRNA-seq by quantitative real-time PCR

Quantitative real-time PCR (qRT-PCR) was carried out on selected candidate genes to technically validate the data generated by sequencing. PCR primers specific to these genes were designed using Primer Premier 5.0 (Applied Biosystems), and the PCR amplification efficiencies of every primer were determined using a standard curve derived from fourfold serial dilution of a pooled cDNA mixture.

Briefly, 1 μg total RNA was reverse-transcribed into cDNA using the PrimeScript RT reagent kit with gDNA Eraser (RR047A, Takara, Japan). All qRT-PCR was carried out using SYBR Green I Master (Roche) on a Roche LightCycler 480 instrument following the manufacturer’s guidelines. The PCR reaction consisted of 10 μl Blue-SYBR-Green mix, 1 μl forward and reverse primers (10 pM/μl), 7 μl distilled water, and 1 μl of cDNA, in a total volume of 20 μl. The thermal cycling conditions were 3 min at 95°, followed by 40 reaction cycles (10 s at 95° and 20 s at 60°).

In order to quantify and normalize the expression data, the ΔCt (Ct of the target gene minus Ct of the reference gene) was calculated using beta-2-microglobulin (*B2M*) as the reference gene, which had been proved to be the preferred endogenous reference gene in the poly I:C stimulated PBMC (Wang *et al.* 2014). Fold changes between poly I:C treat and control of every sample were calculated, and the correlation analysis of the fold change measured by qRT-PCR and RNA-seq data were conducted using the ‘cor.test’ function of R.

### Data availability

Sequencing data has been deposited in the National Center for Biotechnology Information Sequence Read Archive with accession no. of PRJNA301538 (http://www.ncbi.nlm.nih.gov/sra/?term=PRJNA301538).

## Results

### Summary statistics for the RNA-seq data

Using the Illumina paired-end RNA-seq approach, we sequenced the transcriptome of poly I:C-stimulated and control PBMC of piglets derived from two breeds, Landrace and Dapulian, with different disease resistance. In total, we generated 57.09–61.73 million 100 bp paired-end reads per sample. Prior to assembly, the low quality reads, which was 0.18% of the raw reads, were removed. As a result, a total of 703 million cleaned reads of the 12 samples with Q20 > 98% were produced, which represented approximately 25.1 times the size of the pig genome (∼2.8 Gb). The numbers of reads per individual RNA-seq library are provided in Supplemental Material, Table S1.

Out of 703 million fragments, 547 million (77.81%) reads were aligned (< 2 mismatches) to the reference genome, and 516 million (73.40%) had a unique genomic location, of which 77.13–82.84% fell in annotated exons, 9.77–13.69% were located in introns, and the remaining 6.92–9.18% were assigned to intergenic regions. Unmapped or multiposition matched reads were excluded from further analyses. The detailed alignment information is also presented in Table S1.

### Mapping and gene expression of the PBMC transcriptome

Cufflinks was then used to assemble porcine PBMC transcriptome *de novo*. Consequently, 29,962 transcripts were assembled, which belonged to 22,759 Ensembl genes. Out of the 29,962 assembled transcripts, 28,297 transcripts were included in Ensembl pig genome database, while the other 1668 transcripts were potentially novel transcripts (Table S2). Of the 22,759 expressed genes, about half (10,856 genes, 47.69%) had the maximum expression level with FPKM < 1 among the 12 samples. To minimize errors, genes with FPKM value > 1 and expressed in at least two of the 12 individuals were termed as expressed transcripts, and used for subsequent analysis. Consequently, we identified 11,903 (52.30%) expressed genes (Table S3), and further analyses were conducted based on these.

Out of the 11,903 genes, the great majority of them (11,881 genes, 99.82%) were coexpressed in both the poly I:C stimulation and control groups, whereas the other 22 genes (0.18%) were expressed in only one of the two groups (Table S3). Furthermore, compared with the control group, the average gene expression level of PBMC with poly I:C stimulation was higher (72.93 *vs.* 63.42). On the other hand, compared between the two breeds, though similar expression levels were observed in both the control (64.03 *vs.* 62.81) and the poly I:C stimulation (73.39 *vs.* 72.46) groups, a few more genes (44 genes, 0.37%) were observed to be expressed in only one of the two breeds.

### Differential gene expression analysis of PBMC in response to poly I:C stimulation

**Figure 1:**
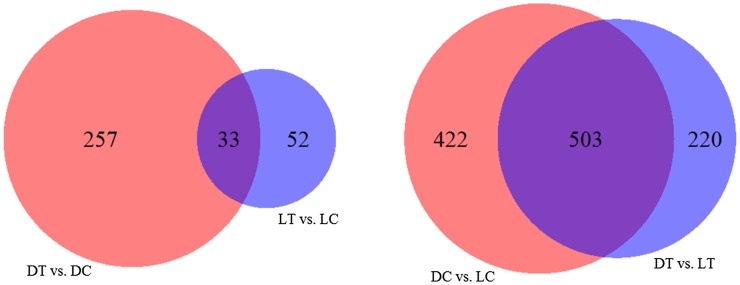
Differentially expressed (DE) genes between poly I:C stimulation group and control group in Dapulian and Landrace (left panel), and DE genes between the two breeds in control groups and poly I:C stimulation groups (right panel). DT, PBMC of Dapulian stimulated with poly I:C for 24 hr; DC, PBMC of Dapulian *in vitro* cultured for 24 hr; LT, PBMC of Landrace stimulated with poly I:C for 24 hr; and LC, PBMC of Landrace cultured *in vitro* for 24 hr.

Using Cuffdiff, the differential gene expression profile of PBMC between the poly I:C stimulation group and the control group was examined for the two breeds separately. Genes with FDR adjusted q-value < 0.05 and log2 (fold-change) > 1 or < −1 were selected as differentially expressed (DE) genes. The summary and complete lists of DE genes of PBMC within the two populations are listed in the Figure 1 (left panel) as well as in Table S4. In Dapulian, there were 290 significantly DE genes detected, of which 231 genes were upregulated and 59 downregulated. In Landrace, only 85 significantly DE genes were observed, with 39 upregulated and 46 downregulated. Furthermore, comparing the two DE gene lists, they shared 33 significantly common DE genes, including 17 upregulated and 16 downregulated genes; detailed information on these common DE genes is shown in Table 1.

**Table 1:** Detailed information of the 33 common significantly DE genes in response to poly I:C stimulation in Dapulian and Landrace

Ensembl ID	Gene Name	Description	Dapulian			Landrace		
			DT (FPKM)	DC (FPKM)	FDR q-Value	LT (FPKM)	LC (FPKM)	FDR q-Value
ENSSSCG00000009644	*ADAM28*	ADAM metallopeptidase domain 28	21.32	48.24	0.0017	3.42	12.34	0.0069
ENSSSCG00000004136	*AIG1*	Androgen-induced 1	4.59	10.52	0.0338	1.03	3.97	0.0289
ENSSSCG00000003491	*AKR7L*	Aldo-keto reductase family 7-like	36.77	89.68	0.0017	11.75	26.66	0.0069
ENSSSCG00000001849	*APN*	Sus scrofa alanyl (membrane) aminopeptidase (ANPEP)	3.57	1.05	0.0017	11.51	4.66	0.0069
ENSSSCG00000008963	*AREG*	Sus scrofa amphiregulin (AREG)	17.14	2.34	0.0017	17.11	7.39	0.021
ENSSSCG00000003524	*C1QA*	Complement component 1, q subcomponent, A chain	15.94	41.53	0.0017	40.25	83.69	0.0236
ENSSSCG00000003525	*C1QC*	Complement component 1, q subcomponent, C chain	8.13	24.69	0.0017	20.96	45.72	0.0069
ENSSSCG00000030638	*CH242-168I5.2*	Uncharacterized protein	6.44	15.62	0.0147	0.88	3.16	0.0422
ENSSSCG00000014310	*CXCL14*	Chemokine (C-X-C motif) ligand 14	3.94	10.22	0.0084	2.30	7.23	0.0326
ENSSSCG00000001469	*DMB*	Sus scrofa MHC class II, DM beta (SLA-DMB)	105.49	224.38	0.0017	42.85	115.39	0.0069
ENSSSCG00000006001	*ENPP2*	Ectonucleotide pyrophosphatase/ phosphodiesterase 2	3.64	0.75	0.0017	10.90	4.66	0.0069
ENSSSCG00000013370	—	Serum amyloid A protein	95.22	12.28	0.0017	74.28	22.12	0.0069
ENSSSCG00000027404	—	Uncharacterized protein	3.98	10.25	0.0017	0.47	2.19	0.0118
ENSSSCG00000028331	*IL1R2*	Sus scrofa interleukin 1 receptor, type II (IL1R2)	305.85	82.71	0.0017	409.61	188.18	0.0165
ENSSSCG00000015617	*G0S2*	G0/G1switch 2	98.52	20.48	0.0017	108.21	52.79	0.0118
ENSSSCG00000002814	*GPR97*	G protein-coupled receptor 97	3.86	0.79	0.0017	28.79	11.27	0.0069
ENSSSCG00000008939	*IGJ*	Immunoglobulin J polypeptide, linker protein for immunoglobulin alpha and mu polypeptides	220.74	80.51	0.0017	61.53	30.65	0.0342
ENSSSCG00000030825	*IGLV-9*	Uncharacterized protein	344.37	158.82	0.0017	154.47	75.41	0.0342
ENSSSCG00000008162	*IL1R1*	Interleukin 1 receptor, type I	15.71	1.94	0.0017	34.97	16.07	0.0069
ENSSSCG00000008163	*-*	Uncharacterized protein	350.74	84.15	0.0017	450.17	211.98	0.0118
ENSSSCG00000012066	*KCNJ15*	Potassium inwardly rectifying channel, subfamily J, member 15	6.68	0.83	0.0017	24.23	8.18	0.0069
ENSSSCG00000000688	*LAG3*	*Sus scrofa* lymphocyte-activation gene 3 (LAG3)	7.24	2.64	0.0017	5.97	2.29	0.0236
ENSSSCG00000026478	*PADI2*	Uncharacterized protein	1.63	5.14	0.0017	0.56	1.54	0.0118
ENSSSCG00000025644	*PCD1A*	*Sus scrofa* CD1 antigen (CD1.1)	71.32	163.80	0.0017	18.29	71.18	0.0069
ENSSSCG00000006451	*PCD1B*	*Sus scrofa* CD1B antigen (PCD1B)	3.77	9.82	0.0084	0.80	5.78	0.0069
ENSSSCG00000005688	*PTGES*	*Sus scrofa* prostaglandin E synthase (PTGES)	15.16	0.57	0.0017	17.68	7.85	0.0342
ENSSSCG00000013369	*SAA1*	*Sus scrofa* serum amyloid A protein (LOC100525856)	72.61	12.15	0.0017	406.62	68.56	0.0069
ENSSSCG00000028525	*SAA4*	*Sus scrofa* serum amyloid A2 (LOC733603)	1024.97	111.13	0.0017	2757.84	958.33	0.0257
ENSSSCG00000003592	*SDC3*	Syndecan 3	14.75	31.95	0.0017	4.95	17.54	0.0069
ENSSSCG00000004889	*SERPINB10*	Serpin peptidase inhibitor, clade B (ovalbumin), member 10	25.66	54.74	0.0017	1.48	5.63	0.0069
ENSSSCG00000002032	*SLC7A8*	Solute carrier family 7 (amino acid transporter light chain, L system), member 8	13.63	41.49	0.0017	2.11	16.67	0.0069
ENSSSCG00000026043	*TGM3*	Transglutaminase 3	521.85	30.04	0.0017	664.19	193.37	0.0069
ENSSSCG00000001614	*TREM2*	Uncharacterized protein	29.88	60.92	0.0181	0.84	13.70	0.021

DT, PBMC of Dapulian stimulated with poly I:C for 24 hr; DC, PBMC of Dapulian *in vitro* cultured for 24 hr; LT, PBMC of Landrace stimulated with poly I:C for 24 hr; LC, PBMC of Landrace cultured *in vitro* for 24 hr.

GO enrichment analysis using the topGO package demonstrated that a total of 271 significant enriched GO terms were observed after FDR multiple test adjustment (FDR < 0.05) for the DE genes detected in Dapulian (Table S5), including 243 terms for biological process (BP) category, 16 terms for molecular function category (MF), and 12 terms for cellular component category (CC). For BP category, most of the terms were involved in immune-related process, such as ‘immune system process’, ‘inflammatory response’, ‘defense response’, and ‘regulation of defense response’. For molecular function (MF), some of the terms were related with activity of cytokine, chemokine and growth factor, and some with receptor binding or activity, such as cytokine/chemokine receptor binding, G-protein coupled receptor binding, and transmembrane signaling receptor activity. For cellular component (CC), the top three terms were related with extracellular space, extracellular region, extracellular region part, and the other involved different cellular components. On the other hand, due to the small number of DE genes detected in Landrace, only 13 significantly enriched GO terms were observed (Table S5), including four BP terms (immune system process, acute-phase response, immune response, cell chemotaxis), five MF terms (cargo receptor activity, receptor activity, interleukin-1 receptor activity, carbohydrate binding, interleukin-1, Type II, blocking receptor activity), and four CC terms (extracellular space, extracellular region, cell surface, extracellular region part). Out of the 13 significantly enriched GO terms in Landrace, nine overlapped with those of Dapulian; detailed information on these overlapped GO terms is presented in Table 2.

**Table 2:** Common GO terms in response to poly I:C stimulation in Dapulian and Landrace

GO.ID	Term	Dapulian				Landrace			
		Annotated Gene Number	Significant	Expected	FDR q-Value	Annotated Gene Number	Significant	Expected	FDR q-Value
GO_BP:0002376	Immune system process	878	80	26.13	1.79E-17	878	23	7.67	7.25E-03
GO_BP:0006953	Acute-phase response	11	6	0.33	7.89E-05	11	4	0.1	8.94E-03
GO_BP:0006955	Immune response	445	54	13.24	5.15E-16	445	15	3.89	1.72E-02
GO_BP:0060326	Cell chemotaxis	95	21	2.83	6.52E-10	95	7	0.83	4.47E-02
GO_MF:0004872	Receptor activity	345	27	9.46	2.72E-04	345	13	2.77	4.04E-03
GO_MF:0004908	Interleukin-1 receptor activity	5	4	0.14	8.36E-04	5	3	0.04	4.55E-03
GO_CC:0005615	Extracellular space	352	56	10.36	9.63E-24	352	17	3.16	9.25E-06
GO_CC:0009986	Cell surface	261	21	7.68	6.34 E-03	261	12	2.34	1.18E-03
GO_CC:0044421	Extracellular region part	1528	85	44.98	1.06E-07	1528	28	13.7	1.71E-02

To provide further insight into the gene expression profile, we also performed GSEA on the Dapulian and Landrace data with the canonical pathways of the C2 catalog of functional gene sets. GSEA is a computational method that determines *a priori* whether a defined set of genes shows statistically significant differences in expression between two biological states, and has proved successful in discovering molecular pathways involved in human diseases (Mootha *et al.* 2003; Subramanian *et al.* 2005). We identified a large number (118) of significantly enriched genes sets between the poly I:C stimulation (DT) group and the control (DC) group of Dapulian. On the other hand, we found 21 significantly enriched genes sets between the poly I:C stimulation (DT) group and control (DC) group of Landrace (Table S6). Further analysis indicated that 15 (71.43%) of the 21 enrichment gene sets were consistent with the top enriched gene sets of Dapulian. The information of the 15 common enriched gene sets is detailed in Table 3. In particular, 11 of the 15 common gene sets gave a consistent picture of immune response-related ones, including eight cytokine-related gene sets (cytokine-cytokine receptor interaction, Jak-STAT signaling pathway, cytokine network, signal transduction through IL1R, cytokines and inflammatory response, TNFR2 signaling pathway, IL12-mediated signaling events, and IL27-mediated signaling events) and three T-cell-related gene sets (the 4-1BB-dependent immune response, selective expression of chemokine receptors during T-cell polarization and Th1/Th2 differentiation).

**Table 3:** Common gene sets in response to poly I:C stimulation in Dapulian and Landrace

Name	Brief Description	Dapulian		Landrace	
		NES	FDR q-Value	NES	FDR q-Value
BIOCARTA_IL1R_PATHWAY	Signal transduction through IL1R	2.0486	4.18E-03	2.0162	1.01E-02
BIOCARTA_INFLAM_PATHWAY	Cytokines and inflammatory response	1.9446	1.02E-02	2.2691	4.36E-04
BIOCARTA_CYTOKINE_PATHWAY	Cytokine network	1.9291	1.12E-02	1.9777	1.58E-02
BIOCARTA_TH1TH2_PATHWAY	Th1/Th2 differentiation	1.8849	1.67E-02	1.9921	1.34E-02
BIOCARTA_TNFR2_PATHWAY	TNFR2 signaling pathway	1.8226	2.83E-02	1.8860	3.22E-02
BIOCARTA_NKT_PATHWAY	Selective expression of chemokine receptors during T-cell polarization	1.7904	3.06E-02	1.9009	2.86E-02
BIOCARTA_41BB_PATHWAY	The 4-1BB-dependent immune response	1.7509	4.06E-02	1.8876	3.29E-02
KEGG_JAK_STAT_SIGNALING_PATHWAY	Jak-STAT signaling pathway	2.3493	0.00E+00	2.3095	1.31E-03
KEGG_CYTOKINE_CYTOKINE_RECEPTOR_INTERACTION	Cytokine-cytokine receptor interaction	2.3249	0.00E+00	2.0778	4.79E-03
PID_FOXM1PATHWAY	FOXM1 transcription factor network	2.2466	8.97E-04	2.1506	2.29E-03
PID_IL12_2PATHWAY	IL12-mediated signaling events	1.9868	6.67E-03	2.3045	6.54E-04
PID_AP1_PATHWAY	AP-1 transcription factor network	1.7857	3.05E-02	2.1452	2.33E-03
PID_IL27PATHWAY	IL27-mediated signaling events	1.7240	4.64E-02	2.0932	4.53E-03
REACTOME_PEPTIDE_LIGAND_BINDING_RECEPTORS	Genes involved in peptide ligand-binding receptors	1.8657	2.05E-02	1.9269	2.13E-02
REACTOME_DEGRADATION_OF_THE_EXTRACELLULAR_MATRIX	Genes involved in degradation of the extracellular matrix	1.8005	2.95E-02	1.9371	2.13E-02

NES, normalized enrichment score.

### Differential gene expression analysis of PBMC between breeds

In order to identify the breed difference in gene expression, using the same method and criteria as analysis in response to poly I:C stimulation, we also compare the gene expression profile of PBMC between the two breeds, including the control groups (DC *vs.* LC) and poly I:C stimulation groups (DT *vs.* LT) separately. The summary and list of DE genes are provided in Figure 1 (right panel) and Table S7. For the control groups, *i.e.*, PBMC cultured only for 24 hr, 925 genes were differentially expressed, of which 429 genes were upregulated and 496 downregulated. On the other hand, for the poly I:C stimulated groups, 723 DE genes were observed, with 431 upregulated and 292 downregulated. Comparing the two DE gene lists, there was a substantial overlap (503 genes), including 279 upregulated and 224 downregulated genes.

A large number of significant GO terms (Table S8) were enriched for the DE genes of the control group (374) and the poly I:C stimulation group (304). Similar to those of DE genes in response to poly I:C stimulation, most of the GO terms were immune-related. However, outwith those immune-related terms, there were some additional themes around development and growth of various tissues, such as anatomical structure morphogenesis, regulation of developmental process, muscle structure development, and regulation of fat cell differentiation.

For gene set enrichment analysis by GSEA, only 15 significant gene sets were detected between the control groups of the two breeds, and one significant gene set between the stimulation group of the two breeds. Detailed information on these significant gene sets is listed in Table S9. It can be seen that most of the gene sets were immune-related. Similar to GO analysis, other than immune-related functions, there were some other themes, such as HIF-2-alpha transcription factor network, genes involved in respiratory electron transport, and fatty acid metabolism.

### Quantitative real-time PCR validation

**Figure 2:**
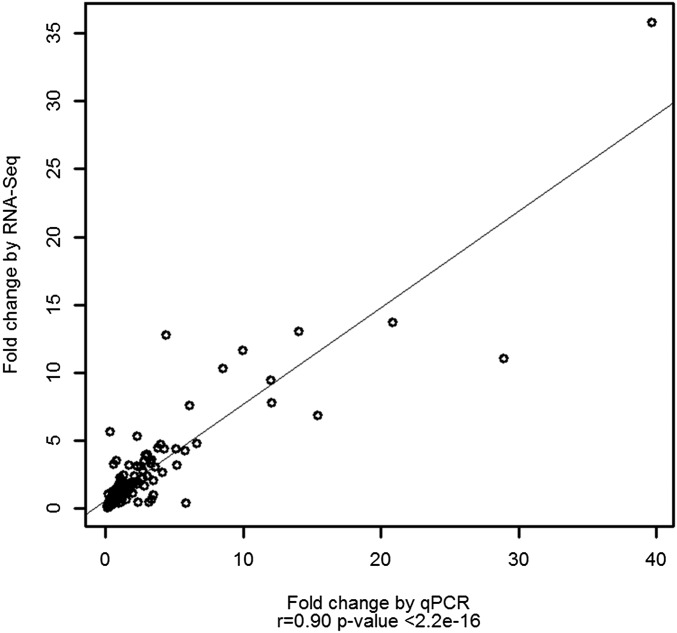
Correlation analysis of the fold change between qRT-PCR and RNA-seq.

In order to technically validate the data generated by sequencing, 23 genes were selected for validation using qRT-PCR. For each of the 23 genes selected, one pair of specific qPCR primers was designed, as detailed in Table S10. PCR amplification efficiencies of every primer ranged from 0.9 to 1.1, as determined using a standard curve derived from a pooled cDNA mixture. All raw data and statistical results of these qRT-PCR tests are available in Table S11. Most of validated genes were differentially expressed in the RNA-seq, while some were not but have an important biological function. So, instead of comparing the significant p-value, in the study, correlation analysis of the fold change between qRT-PCR and RNA-seq was conducted to compare the consistency of the two techniques. As demonstrated in Figure 2 and Table S12, the fold changes measured by RNA-seq and qRT-PCR were significantly correlated (p < 2.2e–16) with a correlation coefficient of 0.9. This result demonstrated the high reliability and accuracy of RNA-seq gene expression data in this study.

## Discussion

In this study, we performed a genome-wide comparative analysis of the mRNA transcriptome between poly I:C-treated and untreated PBMC samples. The results showed that poly I:C challenge can stimulate 290 and 85 significantly DE genes in Dapulian and Landrace, respectively. These DE genes were enriched mainly in immune-related GO terms, such as immune system process, acute-phase response, immune response, and cell chemotaxis. Our results demonstrated the PBMC samples challenged with poly I:C can elicit certain gene expression changes involved in immune response. The method we have applied herein, which does not require pathogen challenge of the animal, could potentially permit *in vitro* screening of animals for optimal innate immune responsiveness. Furthermore, the culture system has the advantage that it largely eliminates the effect of the *in vivo* environment, and provides a convenient basis for analysis of the genetic variation in host pathogen interaction using *in vitro* challenge models.

Dapulian, an indigenous pig breed in North China, exhibits many different characteristics from modern commercial breeds, in growth performance, meat quality, and disease resistance, especially to porcine reproductive and respiratory syndrome (PRRS) (Jiang *et al.* 2013; Xing *et al.* 2014). However, the genetic basis and mechanisms underlying these differences are not clear. DE genes in response to poly I:C stimulation in different breeds may be possible factors leading to the difference in response to viral diseases, and might be exploited in breeding for increased disease resistance or tolerance. Consequently, we compared the difference in DE genes in response to poly I:C stimulation in the two breeds, Dapulian and Landrace. As a result, we detected more DE genes in Dapulian than Landrace (290 *vs.* 85), indicating that Dapulian pigs have a stronger immune response to poly I:C challenge than Landrace pigs. The different characteristics of the two breeds may be the most likely reason for the different DE genes identified in the two breeds. Variability in gene expression of the same tissues in different breeds has been demonstrated by other studies (Gao *et al.* 2010; Sodhi *et al.* 2014). Other factors, such as the small samples (three piglets per breed), and substantial interindividual variation between pigs within breeds, would also lead to different DE genes. Larger samples are needed to further validate the DE genes. Although differences in the DE genes existed, as expected, the two breeds shared some common significantly DE genes (*n* = 33). Among these, most are important genes involved in immune response, such as *LAG3*, *SAA1*, *IL1R1*, *DMB*, and *IGJ*, which may play crucial rules in the immune response of poly I:C stimulation.

Next-generation sequencing, as well as microarrays, generate measurements of gene activity at a genomic scale. Analysis of this type of data usually follows one of two approaches. One approach identifies DE genes across phenotypes of interest, and the other, pioneered by GSEA, focuses on coordinated differential expression of annotated groups of genes, or gene sets, and produces results that can be interpreted more easily in terms of the relevant biological processes (Liberzon *et al.* 2015). In the present study, as well as analysing DE genes, we also conducted gene sets analysis using GSEA. Consequently, we also identified a large number of significantly enriched genes sets in Landrace and Dapulian separately, and some of them (g = 15) were shared by the two breeds. The common gene sets, as well as the DE genes, as shown in Table 1 and Table 3, would provide crucial information to help understand the immune regulation of this organism in response to RNA viruses. Similar to DE genes, most of the common gene sets were related to immune response, including eight cytokine-related gene sets, and three T-cell-related gene sets. The consistency makes sense because the major driving force of the enrichment calculation in GSEA is the largely changing genes (Huang *et al.* 2009). Thus, GSEA is complementary to single-gene approaches, and provides a framework with which to examine changes operating at a higher level of biological organization (Mootha *et al.* 2003; Subramanian *et al.* 2005). In addition, from the perspective of individual genes, 33 out of the 85 DE genes (38.82%) of Landrace can be shared by Dapulian, whereas, from the perspective of gene sets, 15 out of the 21 (71.43%) of the enriched gene sets of Landrace are shared by Dapulian. Therefore, gene set analysis using GSEA showed much greater consistency across the two breeds than single-gene analysis.

Toll-like receptors (TLRs) are the most important class of innate pattern recognition receptors (PRRs), which can recognize the conserved patterns derived from microbial pathogens identified as PAMPs. Of the multiple types of TLRs, *TLR3* is implicated in the recognition of viral dsRNA (Takeda and Akira 2005), and has been shown to be upregulated in human PBMC (Huang *et al.* 2006), as well as in mast cells (Kulka *et al.* 2004), after poly I:C stimulation. However, further study indicated that *TLR3* was the immediate early responding gene, and was upregulated at 3 hr post poly I:C stimulation, whereas there was no significant difference at 12 hr post stimulation (Huang *et al.* 2006). In our results, we observed no significant upregulation in *TLR3*, which is consistent with the fact that PBMC were stimulated for 24 hr. Previous studies also demonstrated that poly I:C induced activation of NF-κβ (Alexopoulou *et al.* 2001; McCartney *et al.* 2009), which functions as a transcription factor to regulate the expression of genes influencing a broad range of biological processes (Hayden and Ghosh 2008). In our DE genes, we also found a large set of NF-κβ target genes, for instance, cytokines, such as *TNF*, *IL6*, *IL10*, *IL1A*, and *IL12A*; chemokines, such as *CCL2*, *CCL4*, *CXC5*, and *CCL20*; and transcription factors and their modulators, such as *NFKBIA*, *NFKBIZ*, *TNFAIP3*, *IER3*, and *LGALS3*.

Besides the difference between the two breeds in response to poly I:C stimulation, we also compared the difference in the two breeds, including difference in their control groups and poly I:C stimulation groups, separately. Consequently, we identified large sets of DE genes and many gene sets in both control groups and poly I:C stimulation groups. Similar to the response to poly I:C stimulation, these DE genes and gene sets are mainly immune-related. PBMC is the main immune cell in whole blood, and it is reasonable that the DE genes between breeds are involved mainly in the immune response. However, PBMC are, to some extent, a heterogeneous mixture of peripheral blood leukocytes, and play multiple other roles besides immune response. Consequently, in addition to immune-related GO terms or gene sets, there were certain additional themes around development and growth of various tissues, which may be correlated with the different characteristics of the two breeds. For instance, there were significant GO terms, such as structure morphogenesis, regulation of developmental process, muscle structure development and regulation of fat cell differentiation, and gene sets, such as HIF-2-alpha transcription factor network, genes involved in respiratory electron transport, and fatty acid metabolism. Specifically, there were 26 genes included in the gene set of KEGG_FATTY_ACID_METABOLISM (Table S9), the top 15 genes belong to the leading gene subset, which have significantly different expression levels between the two breeds.

Previous studies indicated that TLRs, and other pattern recognition receptors, are highly polymorphic in pigs (Kojima-Shibata *et al.* 2009; Bergman *et al.* 2010). We speculate that it is possible that they are expressed differentially between breeds. Indeed, we detected several TLRs that are significantly upregulated in Dapulian, especially *TLR6* and *TLR8*, which were significantly upregulated in Dapulian in both the control groups and the stimulation groups. Furthermore, between the breeds, we also identified a large set of other DE genes that play crucial roles in the immune response, such as chemokines, C-type lectins, and interferon-induced proteins. These DE genes may play important roles in the disease-resistant difference between the two breeds.

In summary, our study confirmed that PBMC samples challenged with poly I:C can elicit certain gene expression changes, similar to those induced by RNA viruses, proving that PBMC represent an attractive tissue source for a variety of applications. This study, being the first global expression profiling of poly I:C stimulated porcine PBMCs, has detected and characterized a large set of DE genes and enriched gene sets of PBMC in response to poly I:C stimulation, and between the two breeds, which could provide crucial information to help understand the immune regulation of this organism towards RNA viruses, and molecular mechanisms of differential genetic resistance to viral infection in modern and indigenous pigs.

## Supplementary Material

Supplemental Material
