# Supplementary material for: Transcriptomic Analysis Identifies Candidate Genes and Gene Sets Controlling the Response of Porcine Peripheral Blood Mononuclear Cells to Poly I:C Stimulation
Source: G3 (Bethesda). 2016 Feb 29;6(5):1267–75. doi: 10.1534/g3.116.028290 (PMC4856078; doi:10.1534/g3.116.028290)
Supplement: Supplemental Material [file supp_6_5_1267__index.html]

Transcriptomic Analysis Identifies Candidate Genes and Gene Sets Controlling the Response of Porcine Peripheral Blood Mononuclear Cells to Poly I:C Stimulation — Supplemental Material 

# Transcriptomic Analysis Identifies Candidate Genes and Gene Sets Controlling the Response of Porcine Peripheral Blood Mononuclear Cells to Poly I:C Stimulation

## Supplemental Material for Wang *et al.*, 2016

**Files in this Data Supplement:**

- Table S1 - Summary of the sequencing data. (.xlsx, 10 KB)
- Table S2 - Novel transcripts identified in PBMC. (.xlsx, 2181 KB)
- Table S3 - Detail information of the 11,903 genes with FPKM > 1 and expressed in at least 2 samples. (.xlsx, 2937 KB)
- Table S4 - Detailed information of DE genes between poly I:C stimulation group and control group in Dapulian (A) and Landrace (B). (.xlsx, 73 KB)
- Table S5 - Significantly enriched GO terms of DE genes between poly I:C stimulation group and control group in Dapulian (A) and Landrace (B). (.xlsx, 29 KB)
- Table S6 - Significantly enriched genes sets in expression profile of PBMC between poly I:C stimulation group and control group in Dapulian (A) and Landrace (B). (.xlsx, 23 KB)
- Table S7 - Detailed information of DE genes between control groups of Dapulian and Landrace (A) and poly I:C stimulation groups of Dapulian and Landrace (B). (.xlsx, 252 KB)
- Table S8 - Significant GO terms of DE genes between control groups of Dapulian and Landrace (A) and poly I:C stimulation groups in Dapulian and Landrace (B). (.xlsx, 54 KB)
- Table S9 - Genes sets significantly enriched in expression profile of PBMC between the control groups of Dapulian and Landrace (A) and poly I:C stimulation groups in Dapulian and Landrace (B). (.xlsx, 11 KB)
- Table S10 - Detailed information of primers used in qRT-PCR assay. (.xlsx, 11 KB)
- Table S11 - Detailed information of ΔCt measured by qRT-PCR. (.xlsx, 13 KB)
- Table S12 - Fold change between poly I:C stimulation and control PBMC per sample measured by qRT-PCR and RNA-seq. (.xlsx, 13 KB)
